# Phosphatidylcholine Alteration Identified Using MALDI Imaging MS in HBV-Infected Mouse Livers and Virus-Mediated Regeneration Defects

**DOI:** 10.1371/journal.pone.0103955

**Published:** 2014-08-07

**Authors:** Eun-Sook Park, Jeong Hwa Lee, Ji Hye Hong, Yong Kwang Park, Joon Won Lee, Won-Jae Lee, Jae Won Lee, Kwang Pyo Kim, Kyun-Hwan Kim

**Affiliations:** 1 Department of Pharmacology and Center for Cancer Research and Diagnostic Medicine, IBST, School of Medicine, Konkuk University, Seoul, Korea; 2 KU Open Innovation Center, Konkuk University, Seoul, Korea; 3 Research Institute of Medical Sciences, Konkuk University, Seoul, Korea; 4 Department of Applied Chemistry, Kyung Hee University, Yongin, Gyeonggi, Korea; SRI International, United States of America

## Abstract

In this study, we investigated whether hepatitis B virus (HBV) causes the alteration of lipid metabolism and composition during acute infection and liver regeneration in a mouse model. The liver controls lipid biogenesis and bile acid homeostasis. Infection of HBV causes various liver diseases and impairs liver regeneration. As there are very few reports available in the literature on lipid alterations by HBV infection or HBV-mediated liver injury, we have analyzed phospholipids that have important roles in liver regeneration by using matrix-assisted laser desorption/ionization (MALDI)-imaging mass spectrometry (IMS) in the livers of HBV model mice. As a result, we identified different phosphatidylcholines (PCs) showing significant changes in their composition as well as cationized ion adduct formation in HBV-infected mouse livers which are associated with virus-mediated regeneration defects. To find the factor of altered PCs, the expression kinetics of enzymes was also examined that regulate PC biosynthesis during liver regeneration. It is noteworthy that the expression of choline-phosphate cytidylyltransferase A (PCYT1A) was significantly delayed in wild type HBV-expressing livers. Moreover, the amount of hepatic total PC was also significantly decreased in wt HBV-expressing mice. These results suggest that infection of HBV alters the composition of PCs which may involve in HBV-mediated regeneration defects and liver disease.

## Introduction

Lipid metabolism is one of the essential liver functions and the aberrant metabolism of liver has been reported to cause liver diseases such as inflammation and fibrosis [1]. Infection of hepatitis B virus (HBV), which was estimated to affect 400 million people worldwide, also causes a variety of liver diseases including acute or chronic inflammation, cirrhosis, and hepatocellular carcinoma (HCC) [2]–[4]. It is well known that HBV infection provokes disturbance of lipid metabolism; however, little is known about virus-mediated lipid changes.

The liver has a unique ability to be regenerated after injury or partial hepatectomy through relatively well-defined and dynamic processes [5], [6]. Such regenerative potential is essential for survival after acute or chronic liver injury. The hepatic regeneration is a highly complex process that involves variety of growth factors such as hepatocyte growth factor (HGF), interleukin (IL)-6, tumor necrosis factor (TNF)-α, and transforming growth factor (TGF)-β [5]–[10]. Multiple factors can influence regeneration processes by providing stimulatory or inhibitory signals for hepatocyte proliferation.

Liver regeneration after liver damages caused by hepatotoxins such as diet toxins and pathogen infections and surgical resection is critical for liver homeostasis. Since phospholipids are major components of the cell membrane, they have important roles during liver regeneration and lipid metabolism. In this regard, the aberrant accumulation of lipid and triglycerides were observed during liver regeneration after partial hepatectomy [11]–[13]. Lipids that accumulate during liver regeneration are thought to play some transient roles for the liver remnant by supplying the energy and membrane components needed for cell division [11], [12]. Moreover, fatty acid composition is altered in liver after partial hepatectomy [14] and especially, the hepatic content of phospholipids is increased [15]. These observations suggest that phospholipid metabolism may be critically associated with the liver regeneration. Recently, we and other groups have reported that HBV infection can impair the liver regeneration through the HBV X protein (HBx) [16]–[20], suggesting that the altered biogenesis of phospholipids, is involved in the regeneration process after the liver injury caused by HBV infection. However, there have been no studies examining the effect of HBV infection on the alterations of phospholipids during infection or liver regeneration.

Currently, matrix-assisted laser desorption ionization (MALDI)-imaging mass spectrometry (IMS) is an established approach for detecting and localizing a large variety of endogenous molecules including drugs and their metabolites, proteins, peptides and phospholipids. In our previous studies, MALDI-IMS was applied to visualize the differential distributions of phospholipids in diseased lesions and adjacent normal regions of surgical tissues including the breast, lung, ovarian, and intrahepatic cholangiocarcinoma cancers as well as focal cerebral ischemia [21]–[25]. In this study, we applied MALDI-IMS to examine the distributions and alterations of phospholipids in livers with HBV infection and HBV-mediated regeneration defects. We showed that the composition of several phosphatidylcholines (PCs) and expression level of an enzyme for PC biosynthesis were significantly altered in livers with HBV infection and HBV-mediated regeneration defects.

## Materials and Methods

### Mouse model for HBV infection

A mouse model for acute HBV infection was used as previously described [19], [26]. Briefly, six- to seven-week-old male BALB/c mice (n = 3 per group) were hydrodynamically injected with 25 ug of plasmid DNA (pGEM-4z, pHBV1.2 (wt), pHBV1.2 (HBx-)) into the tail vein in a volume of phosphate-buffered saline (PBS) equivalent to 10% of the mouse body weight. The total volume of DNA was delivered into the vein with high pressure within 4–6 seconds (hydrodynamic *in vivo* transfection). All procedures involving experimental animals were approved by the Animal Care Committee in Konkuk University.

### Partial hepatectomy

To induce physiological liver regeneration in mice, a model of 70% partial hepatectomy was used as previously described [19], [27]. Briefly, 4 days after hydrodynamic injection with DNA, approximately 70% of the total liver mass was resected after intraperitoneal administration of xylazine (10 ug/kg body weight) and zoletil 50 (10 mg/kg body weight). After anesthetization, the left lobe and a part of the median lobe of each mouse liver were removed through a mid-abdominal incision. After suturing the abdominal wall, the animals were returned to their cages and permitted to feed *ad libitum*. Liver regeneration was evaluated by the ratio of body weight and the liver weight 8 days after partial hepatectomy.

### HBV antigen analysis and histological examination of mouse tissue

Surface antigen analysis was performed with mouse serum using the HBsAg detection enzyme-linked immunosorbent assay (ELISA) kit (Enzygnost HBsAg 5.0, Siemens, Erlangen, Germany). Liver tissues were fixed in 4% formaldehyde, embedded in paraffin and sectioned using a microtome. Paraffin sections were stained with hematoxylin and eosin (H&E) and examined using a microscope.

### Proliferation assay of mouse hepatocytes

To evaluate the proliferation status of mouse hepatocytes after partial hepatectomy, the Ki67-labeling index was monitored as a proliferation marker. Ki67-stained hepatocytes were identified on 4-um tissue sections of paraffin-embedded liver samples according to the manufacturer's instructions (Cell Proliferation Kit, Amersham Life Science, Amersham, UK).

### Tissue processing for MALDI analysis

Liver tissues (n = 2) extracted from mouse specimens were flash-frozen in liquid nitrogen and then stored at −80°C until use. The 10-um-thick tissue sections were cut in a temperature ranging from −20°C to −25°C using cryostat (Leica CM-1850, Leica Microsystems Inc., Solms, Germany). For MALDI-IMS experiments, tissue sections were thaw-mounted on indium-tin-oxide (ITO)-coated glass slides (HST Global, Inc., Hampton, VA, USA). The tissues were then frozen; incubated at 4°C, and at room temperature. Finally, the tissues were dried in a desiccator. For MALDI-MS profiling experiments, a binary matrix solution was prepared by dissolving 3.5 mg each of DHB and CHCA into 1 ml of 70% methanol containing 0.1% TFA as described previously [28], and for the imaging experiments the same matrix was added using ImagePrep (Bruker Daltonics, Billerica, MA, USA). Biological replicates per group were generated from two randomly selected mice.

### MALDI imaging mass spectrometry analysis

Mass spectrometry experiments were performed using an Autoflex III mass spectrometer equipped with smart beam laser technology (Nd:YAG, 355 nm; Bruker Daltonics Inc.). Imaging experiments were performed in both positive and negative ion modes from single tissue section and all ions in the mass-to-charge (*m/z*) ratio range of 500–1,200 at a laser frequency of 200 Hz (3,000 consecutive laser shots) were detected in reflectron mode. For imaging experiments, the data acquisition was under the control of Flex software suite (FlexControl 3.0, FlexImaging 2.1, FlexAnalysis 3.3, Bruker Daltonics) and using a spatial resolution of 150 µm with 500 consecutive laser shots per pixel (50 laser shots per position of a random walk within each pixel). Before each data acquisition, an external calibration was conducted using phospholipid-mixed calibration standards with a mass range of 674–834 Da (positive ion mode) and 564–906 Da (negative ion mode). MALDI LIFT (MS/MS) analysis was directly performed on the tissue after MALDI MS. Different phospholipid species were identified by using LIFT data and lipid database (www.lipidmaps.org).

Clinpro Tools (version 2.2, Bruker Daltonics) and flexImaging software was used for MALDI imaging data processing, and all spectral intensities were divided by the obtained total ion count values. Ion intensities were evaluated by comparing ion pairs from liver sections of different experimental groups after normalization. The Top Hat baseline with a 10% minimal baseline width was used for baseline subtraction. Data reduction was performed at a factor of four.

### Real-time-PCR (RT-PCR)

Whole mouse liver tissues were lysed using Trizol reagent (Invitrogen/Gibco BRL, Carlsbad, CA, USA) according to the manufacturer's instructions, and total RNAs were isolated. Reverse transcription reactions were performed using 2 ug of total RNA and M-MLV reverse transcriptase (iNtRON, Seoul, Korea) in a final reaction volume of 20 ul. The resulting cDNA was PCR-amplified under the following conditions: initial denaturation at 94°C for 5 min, followed by 25–30 cycles of 94°C (30 s), 58°C (30 s), and 72°C (30 s), with a final extension of 5 min at 72°C. The primer sequences used for amplifications were as follows: phosphate cytidylyltransferase 1, choline, alpha isoform (Pcyt1a), forward, 5′-AGGCTACTGTGACCGAGGTA-3′, reverse, 5′-AGTCACCCTGACATAGGGCT-3′; phosphatidylethanolamine N-methyltransferase (Pemt), forward, 5′-GAGAACTCGGAAGCTGAGCA-3′, reverse, 5′-CAGCACAAACACGAATCCCC-3′. Quantitative real-time PCR was performed using a SYBR Green PCR Master Mix (Applied Biosystems, Foster City, CA, USA), and PCR amplification was conducted using the Applied Biosystems (ABI7500) real-time PCR machine. Relative quantification analysis was performed by the comparative ΔΔCt method. The results were presented as an n-fold difference relative to calibrator (RQ = 2^−ΔΔCt^).

### Western blot analysis

Each liver tissue was homogenized in RIPA buffer. Fifty µg of protein was separated by 10–12% SDS-PAGE gel and subjected to Western blot analysis. The primary antibodies used were anti-Pcyt1a (N-20, Santa Cruz Biotechnology, CA, USA) and anti-b-actin (Sigma Aldrich). As secondary antibody, appropriate peroxidase-conjugated secondary antibodies (Santa Cruz Biotechnology, CA, USA) were used. Chemiluminecent detection was done with the ECL Detection Reagent (GE Healthcare, Buckinghamshire, UK) and visualized with a LAS-4000 Luminescent Image analyzer (LAS-4000, Fuji, Tokyo, Japan).

### Quantification of total PC amount

Mice were sacrificed after partial hepatectomy at indicated time points. The amount of total PC was measured by using phosphatidylcholine assay kit (Cell biolabs Inc, San Diego, CA) according to the manufacturer's instruction. At least three mice are used for the analysis of each group.

### Data analysis

Data were obtained by at least 3 independent experiments and the values are presented as the mean ± standard deviation (SD). For MALDI-IMS analysis, two liver tissue samples per groups were independently analyzed. Differences between groups were tested for statistical significance using Student's *t*-test or analysis of variance. The statistical significance was set at p < 0.05.

## Results

### Characterization of mouse model of HBV infection and virus-induced inhibition of liver regeneration

**Figure 1:**
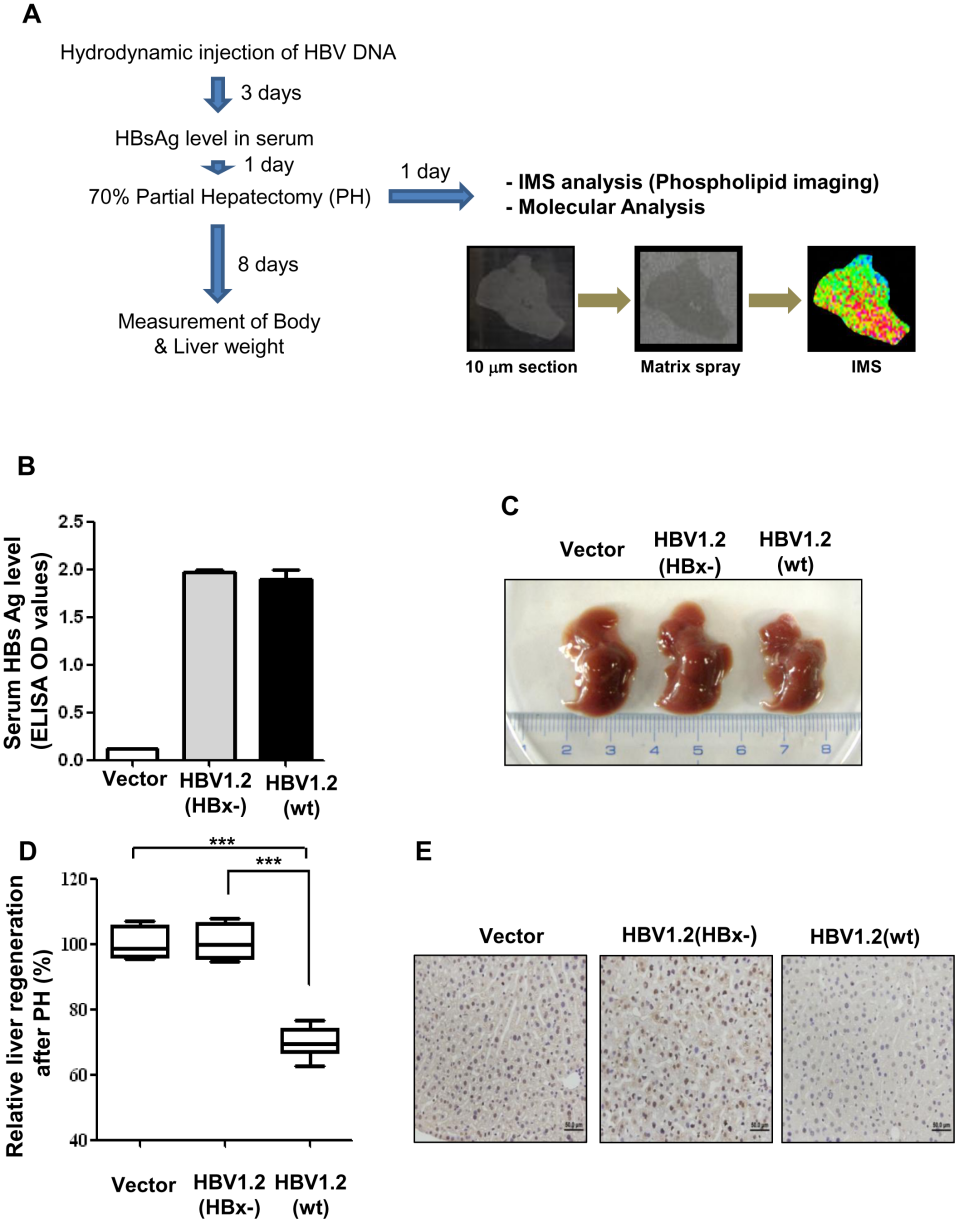
Characterization of HBV mouse model and virus-mediated inhibition of liver regeneration. (A) Overall scheme of experimental procedures. B–E. Validation and characterization of the mouse model of HBV infection used in this study. (B) The HBsAg level in mouse serum at 3 days after hydrodynamic injection. (C) Inhibition of liver regeneration by HBx in HBV mouse model. Macroscopic evaluation of regenerated livers of HBV or HBx mouse model at 8 days after partial hepatectomy. (D) Box plots of liver weight/body weight at 8 days after partial hepatectomy. (E) Ki 67 (marker of cell proliferation) expression in formalin-fixed liver tissues obtained at 48 h after partial hepatectomy. Data are depicted as mean ±S.D. and are representative of three independent experiments. ***, P<0.001 compared with mock vector and HBV1.2 (HBx-) (n = 5∼6 mice per each group).

To investigate whether HBV infection can alter the distribution or composition of phospholipids when regenerating liver using MALDI-IMS, we established a mouse model of HBV infection by hydrodynamically injecting the HBV genome [19], [26]. The overall experimental scheme used in this study is depicted in Figure 1A. To validate whether the HBV mouse could express viral proteins, viral surface protein (HBsAg) was analyzed by ELISA using mice sera at 3 days after hydrodynamic injection. As shown in Figure 1B, both the replication-competent wt HBV (HBV1.2 (wt)) and the HBx-null HBV (HBV1.2 (HBx-)) can express a similar level of HBsAg protein in the serum of HBV infection (Fig. 1B).

More recently, we reported that HBV infection inhibits liver regeneration by epigenetic regulation of urokinase plasminogen activator through HBx [19]. In this study, to correlate the alterations in phospholipid metabolism with HBV-induced liver regeneration defects, we performed phospholipid profiling and imaging with MALDI-MS and MALDI-IMS using an experimental model of the partial hepatectomy after the hydrodynamic injection of wt HBV and HBx-negative HBV. Consistent with the results of our previous study [19], significant inhibition of liver regeneration at 8 days after partial hepatectomy was observed only in wt HBV infection (Fig. 1C and D). We also examined hepatocyte proliferation by counting the Ki67-positive cells (marker of cell proliferation) by immunohistochemical staining. Proliferation of hepatocytes was also markedly reduced in the liver tissues of wt HBV-expressing mice compared to control or HBx-null liver tissues (Fig. 1E). These results collectively suggest that our model is well established for further analysis of phospholipid imaging using MALDI-IMS.

### MS spectra analyses from IMS by PCA and comparison of phospholipid imaging between control and HBV mice in regenerating livers

**Figure 2:**
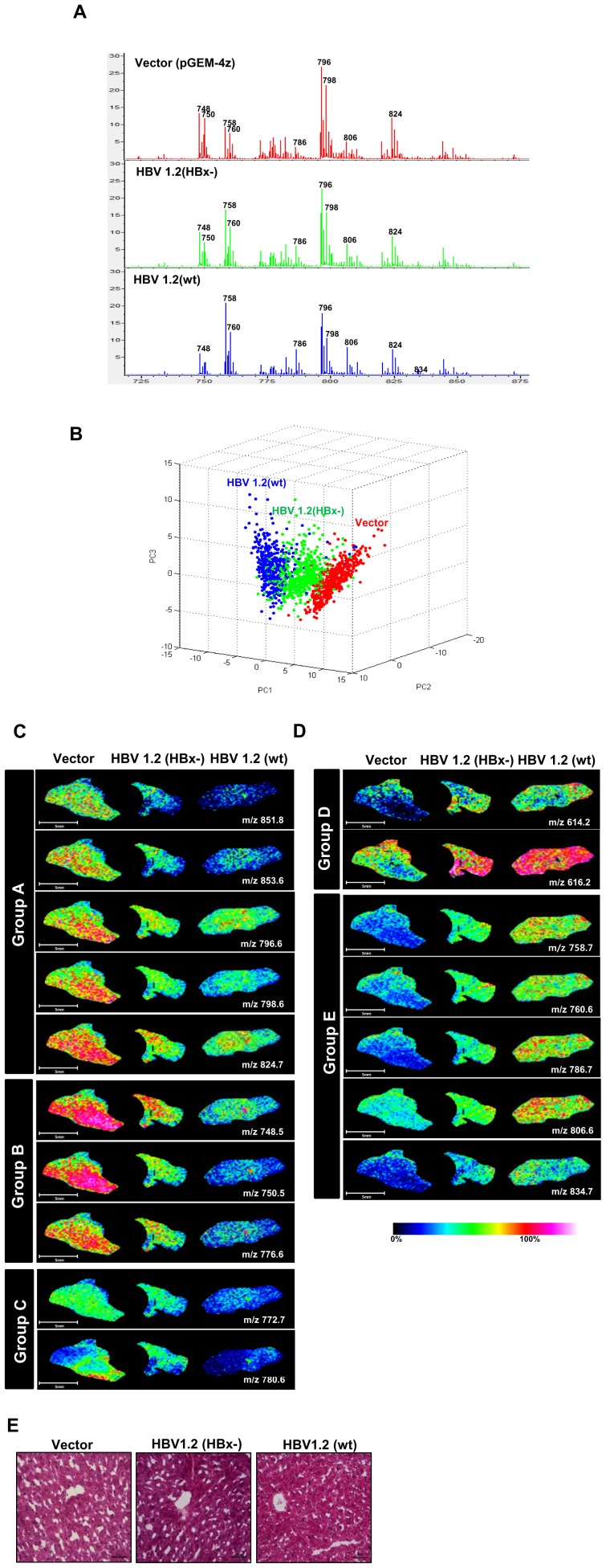
PCA analysis of the averaged MS spectra of phosopholipids from liver tissue sections obtained from 3 different conditions in positive ionization mode and comparison of MALDI images of phospholipids in regenerating liver tissues after partial hepatectomy. (A) Typical MS spectra of liver tissue sections in 3 different conditions obtained from imaging mass spectrometry. (B) PCA analysis of the IMS data acquired in positive-ionization mode. (C, D) Representative MALDI images of molecular ions showing alterations in their levels according to HBV infection with or without HBx expression. (E) H&E-stained liver tissues 24 h after partial hepatectomy. h after partial hepatectomy.

It was previously reported that the content of hepatic phospholipids which are the major components of cell membrane was altered after the partial hepatectomy [15]. Thus, we investigated whether HBV infection could alter the distribution of phospholipids in the regenerating liver using MALDI-IMS. Liver samples (n = 2 per group) extracted from mice of liver regeneration after partial hepatectomy were subjected to MALDI-IMS analysis in both positive and negative modes. Figure 2A shows the averaged MS spectra acquired from whole mouse liver tissue sections in positive ion mode. The spectra show more than 80 peaks (S/N>6) having specific *m/z* values with different relative intensities from different liver tissues under different experimental conditions (Mock, HBV1.2 (HBx-), and HBV1.2 (wt)) (Fig. 2A). The distribution of obtained MS data was also analyzed statistically by principal component analysis (PCA) and classification methods. PCA for total MS spectra was performed from each group using ClinProTools v2.2 (Bruker Daltonics). As a result, PCA provided a visual distinction on the basis of the detected ions according to three different experimental groups as shown in Figure 2B. Interestingly, PCA analysis for averaged MS spectra acquired in negative ion mode showed no significant changes, although more than 55 peaks (S/N>6) were detected (Figure S1).

Of a number of imaging data obtained using MALDI-IMS, we selected 17 ion images acquired in positive ion mode showing significant changes in their abundance in ion images of liver tissue sections from three different groups as shown in Figure 2C and 2D. Ion images from the biological replicates were listed in Figure S2. No significant alterations were observed in ion images in the negative ion mode (data not shown).

To identify the selected 17 ions in Figure 2C and 2D, we performed MS/MS analysis (Figure S3). From the spectra, these ions were identified as seven PCs with three different adducts (H^+^, Na^+^ and K^+^), two sphingomyelins (SMs) and two lyso-phosphatidylcholines (LPCs) as listed in Table 1. Due to the low intensity of m/z 776.6 ion, the structure was not elucidated in these experiments. The phospholipids shown as 17 ion images were classified into 5 groups according to their patterns of level changes. Phospholipids belonging to A, B, and C groups generally showed decreased relative abundances in HBV1.2 (HBx-) and HBV1.2 (wt) compared to the mock, while ion intensities for phospholipids belonging to the D and E groups showed increased relative abundances in HBV1.2 (HBx-) and HBV1.2 (wt) compared to the mock. Phospholipids belonging to group A, SM (42∶2) [M+K]^+^ (m/z 851.8), SM (42∶1) [M+K]^+^ (m/z 853.6), PC (34∶2) [M+K]^+^ (m/z 796.6), PC (34∶1) [M+K]^+^ (m/z 798.6), and PC (36∶2) [M+K]^+^ (m/z 824.7), showed decreased ion intensities in HBV1.2 (HBx-) and HBV1.2 (wt) compared with the mock. Ion intensities of phospholipids belonging to group B, PC (32∶4) [M+Na]^+^ (m/z 748.5), PC (32∶3) [M+Na]^+^ (m/z 750.5) and m/z 776.6 (structure not identified), decreased gradually in the order of the mock, HBV1.2 (HBx-), and HBV1.2 (wt). However, phospholipids of group D, LPC (22∶2) [M+K]^+^ (m/z 614.2) and LPC (22∶2) [M+K]^+^ (m/z 616.2), showed higher ion intensities in HBV1.2 (HBx-) and HBV1.2 (wt) compared with the mock. Ion intensities of phospholipids of group E, PC (34∶2) [M+H]^+^ (m/z 758.7), PC (34∶1) [M+H]^+^ (m/z 760.6), PC (36∶2) [M+H]^+^ (m/z 786.7), and PC (38∶6) [M+H]^+^ (m/z 806.6), increased gradually in the order of the mock, HBV1.2 (HBx-), and HBV1.2 (wt). Interestingly, phospholipids of group C, PC (32∶0) [M+K]^+^ (m/z 772.7) and PC (34∶2) [M+Na]^+^ (m/z 780.6), showed similar ion intensities between the mock and HBV1.2 (HBx-) and the significantly lower ion intensities in HBV1.2 (wt) compared with the mock and HBV1.2 (HBx-).

**Table 1:** Phospholipids showing the alteration in regenerating liver tissues.

Group	*m/z*	Phospholipid Class	FA (Cn: Un)	Adduct
A	851.8	SM	42∶2	K
	853.6	SM	42∶1	K
	796.6	PC	34∶2	K
	798.6	PC	34∶1	K
	824.7	PC	36∶2	K
B	748.5	PC	32∶4	Na
	750.5	PC	32∶3	Na
	776.6	-	-	-
C	772.7	PC	32∶0	K
	780.6	PC	34∶2	Na
D	614.2	LPC	22∶2	K
	616.2	LPC	22∶1	K
E	758.7	PC	34∶2	H
	760.6	PC	34∶1	H
	786.7	PC	36∶2	H
	806.6	PC	38∶6	H
	834.7	PC	40∶6	H

Cn, total chain length; Un, total degree of unsaturation; SM, sphingomyelin;

PC, phosphatidylcholine; LPC, lyso-phosphatidylcholine.

There was no significant difference in liver histology analyzed by H&E staining within the 3 groups (Fig. 2E). IMS data was applied to confirm not only lipid alteration but also the distribution of each ion in the tissue.

### Analysis of potassium cationized adduct formation for selected phospholipids by IMS in regenerating livers

**Figure 3:**
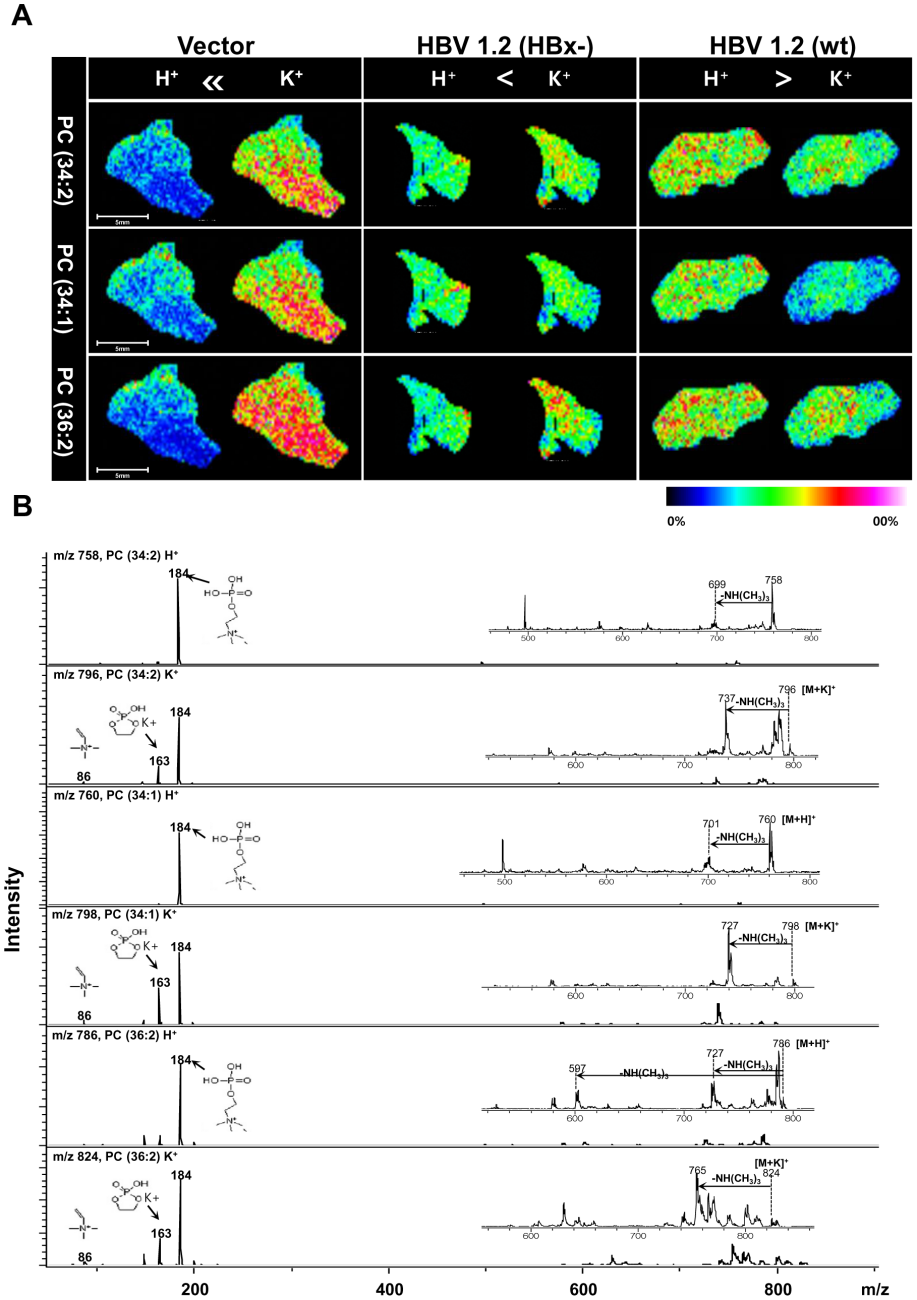
Analysis of selected phospholipids by IMS and MS/MS in regenerating liver tissues. (A) Comparison of the images of H^+^ and K^+^ adduct form of 3 PCs in regenerating liver tissues. (B) Validation of selected PCs [M+H]^+^ and [M+K]^+^ ions by MS/MS analysis.

Interestingly, we observed distinct adduct formation for the most abundant PC species, PC (34∶2), PC (34∶1), and PC (36∶2). Adduct formation of these PCs is different according to the mock and HBV infection with or without HBx expression (Fig. 3). The potassium cationized adducts of these PCs showed higher ion intensities than those protonated forms in the livers of control in which the regeneration is more active than in those of HBV infection. However, in the liver samples of HBV1.2 (HBx-) and HBV1.2 (wt), there was no significant difference in adduct formation. In the case of HBV1.2 (HBx-), the potassium cationized forms of PC (34∶2) (m/z 796.6) and PC (36∶2) (m/z 824.7) showed slightly higher intensities than those protonated forms. In particular, potassium cationized form of PC (34∶1) (m/z 798.6) showed lower intensity than its protonated form in the liver of HBV1.2 (wt) (Fig. 3A). The MS/MS analysis was also applied to assign the formation of protonated form and potassium cationized form of PC (34∶2), PC (34∶1), and PC (36∶2) (Fig. 3B). In the MS/MS data of PCs, the *m/z* 184 ion was mainly detected in its protonated form, whereas the ions at *m/z* 86, *m/z* 163 and *m/z* 184 were detected in their potassium cationized form. From the spectra it is observed that the protonated and potassium cationized PC forms of precursor ions ([M+H]^+^ and [M+K]^+^ ions) and fragment ions mainly show the neutral loss of C_3_H_10_N (59 Da) (Fig. 3B).

It was previously reported that (Na^+^ + K^+^)-stimulated ATPase activity was increased in the hepatic plasma membranes of partially hepatectomized rat [29], [30]. In this study, the high level of potassium cationized form in PCs was observed in the active liver regeneration after partial hepatectomy. However, in the HBV-infected liver, the level of potassium cationized form in PCs was reversed in the liver regeneration.

### Kinetic analysis of major genes involved in PC synthesis in regenerating liver

**Figure 4:**
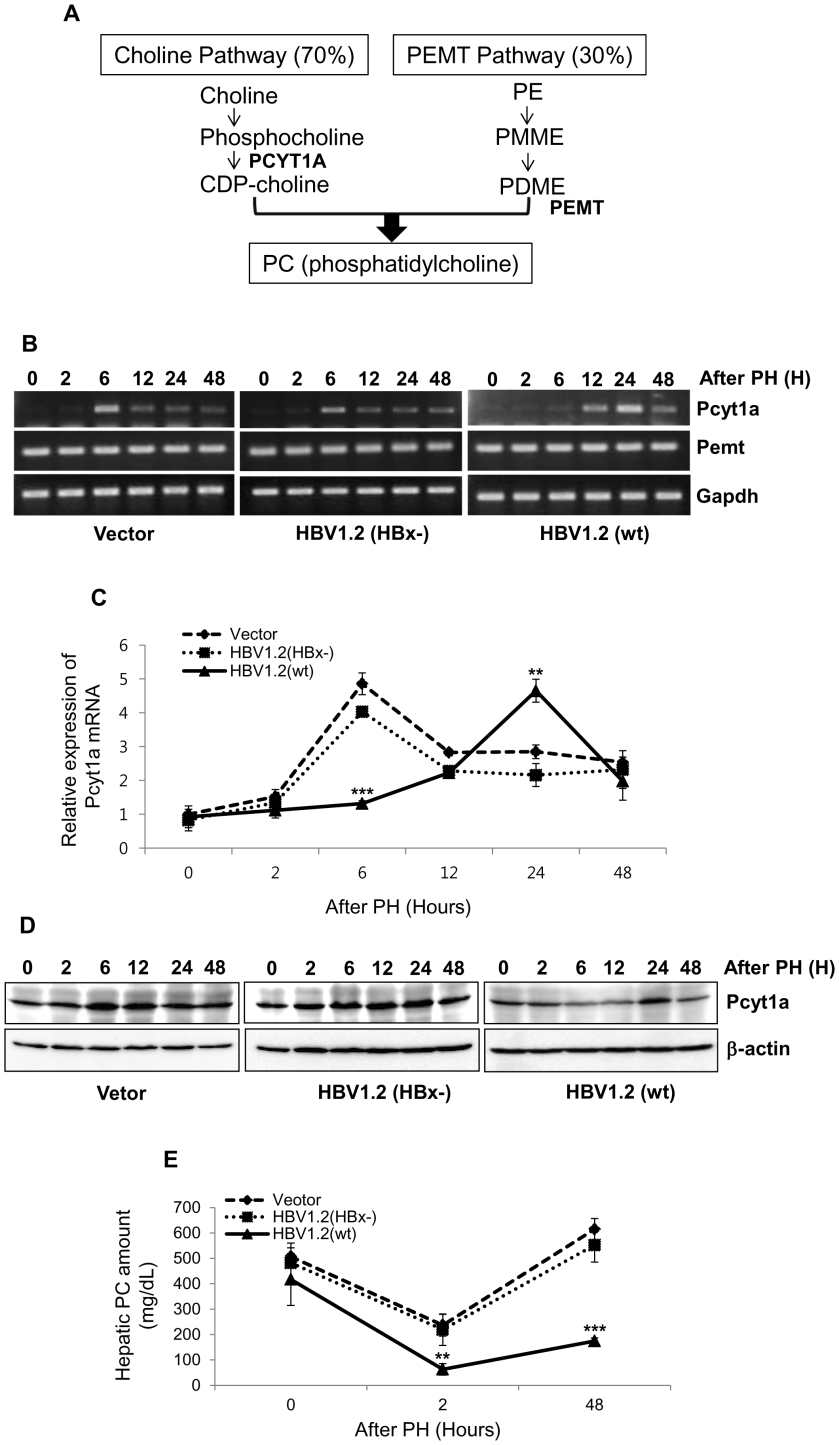
Kinetic analysis of phosphatidylcholines (PCs) synthesis-related genes and measurement of total PCs level in regenerating liver tissues after partial hepatectomy. (A) The schematic pathway of PC biosynthesis. PCYT1A, choline-phosphate cytidylyltransferase A; PE, phosphatidylethanolamine; PMME, triolein-phosphatidyl-N-monomethyl ethanolamine; PDME, triolein-phosphatidyl-N,N-dimethyl ethanolamine; PEMT, phosphatidylethanolamine N-methyltransferase. (B–C) Kinetic analysis of Pcyt1a and Pemt expression in liver tissues after partial hepatectomy. At indicated time intervals after partial hepatectomy, the mRNA expression of indicated genes was analyzed using semi-quantitative RT-PCR (B) and real-time PCR (C), and Western blot analysis (D). (E) Quantification of hepatic PCs level after partial hepatectomy. Data represent the mean ±S.D. and are representative of three independent experiments. **, P<0.01; ***, P<0.001 compared with mock vector and HBV1.2 (HBx-) (n = 3∼4 mice per each group).

The above results demonstrated that PCs are primarily differentially regulated by HBV infection during liver regeneration. Therefore, we investigated whether the expression of genes known to be involved in PC biosynthesis are altered by HBV infection. PC biosynthesis is generally known as being regulated by 2 pathways: the CDP-choline and phosphatidylethanolamine N-methyltransferase (PEMT) pathways [31]. The major enzymes that regulate these 2 pathways include choline-phosphate cytidylyltransferase A (PCYT1A) and PEMT, respectively (Fig. 4A).

To investigate the kinetic changes in these genes in regenerating liver, we determined the mRNA expression level of Pcyt1a and Pemt at several time points after partial hepatectomy. As shown in Figure 4B and 4C, the expression of Pcyt1a was strongly induced at 6 h after partial hepatectomy in the liver of control and HBx-negative HBV mice. However, in the liver of wt HBV infection, the expression level of Pcyt1a was significantly repressed. Interestingly, the expression was induced rather late after partial hepatectomy (24 h). The expression level of Pcyt1a protein was also delayed in the regenerating livers with wt HBV infection (Fig. 4D). To examine the consequence of altered expression of Pcyt1a enzyme, we measured the total amount of hepatic PCs at both early and late time points after partial hepatectomy. As shown in Figure 4E, the level of total PCs was significantly decreased only in the liver of wt HBV infected mice at 48 h after partial hepatectomy. These data demonstrated that the PC biosynthesis was disturbed by wt HBV infection during liver regeneration, suggesting that the regulation of Pcyt1a expression by HBx is involved in the mouse livers with HBV infection and virus-mediated regeneration defects.

## Discussion

In this study, we analyzed the distribution and composition of differentially regulated phospholipids in regenerating livers with HBV infection using MALDI-IMS. Although we could not find significant alterations in phospholipid distribution across the liver tissue, we identified that several PCs altered in the regenerating livers of HBV-infected mice. Moreover, the kinetic changes in genes involved in PC biosynthesis were found in regenerating livers. It was demonstrated that PC biosynthesis is deregulated in the liver of HBV-infected mouse with the virus-mediated regeneration defects through a mechanism mediating the action of HBx protein. Reversely, in livers with HBX-null HBV infection, the recovery of liver regeneration was shown.

Recently, it was reported that the aberrant lipid metabolism mediates liver inflammation and fibrosis [1]. However, it is unclear whether the altered PCs observed in this study resulted from the aberrant lipid metabolism of liver. The chronic infection of HBV undoubtedly induces liver inflammation and fibrosis, indicating that the altered PCs are associated with HBV-related liver diseases.

We identified 17 phospholipids showing significant changes in regenerating liver tissues as shown in Figure 2C and 2D. Phospholipids in A and B groups were altered during HBV infection regardless of HBx expression (Fig. 2C). On the other hand, phospholipids in C, D and E groups showed prominent alterations only in the wt HBV-expressed regenerating liver (Fig. 2D). Phospholipids altered only by wt HBV infection were identified as several PCs. These results suggest that several phospholipids can be altered by HBV infection and it is expected that especially altered PCs (groups C, D, and E) may involve in inhibiting liver regeneration by HBx.

Of phospholipids, several PC represents significant alterations in the HBV 1.2 (wt) compared to the mock and HBV1.2 (HBx-). Thus, we investigated whether the infection of HBV affects PC biosynthesis known to be regulated by 2 pathways: the CDP-choline and phosphatidylethanolamine N-methyltransferase (PEMT) pathways. Several studies have demonstrated that 70% of PC synthesized in the liver is mediated through the CDP-choline pathway, while the remaining 30% is produced by the PEMT pathway [31]. Induction of PC biosynthesis has been shown to be an essential step in cell proliferation in various cells [32]–[34]. Martin *et al*. demonstrated that the amount of PC is increased in regenerating livers and the activity of an enzyme, PCYT1A which involves in PC biosynthesis is significantly enhanced after partial hepatectomy [35]. In this study, we examined the kinetic changes in PC synthesis-related genes in regenerating livers and found that the expression of PCYT1A is clearly elevated at 6 h after partial hepatectomy in control and HBx-negative HBV mice. However, PCYT1A expression was strongly suppressed in wt HBV 1.2 mice early after partial hepatectomy. These data indicate that HBV infection can disturb the expression kinetics of PCs and Pcyt1a through HBx protein.

Several studies have reported that altered liver plasma-membrane fluidity was observed between 15 and 24 h after partial hepatectomy which is associated with the altered composition of phospholipids and functional changes in liver plasma-membrane-bound enzymes [36], [37]. The Na^+^/K^+^-ATPase (Na^+^/K^+^ pump) is located in the plasma membrane of all animal cells and responsible for up to two-third of energy expenditure in a cell. Cells maintain a low concentration of sodium ions and high levels of potassium ions within the cell in order to maintain the cell membrane potential. The (Na^+^/K^+^)-stimulated ATPase activity has shown to be increased in hepatic plasma membranes of partially hepatectomized rat [29], [30]. In this study, the potassium cationized adduct forms of PC (34∶2), PC (34∶1) and PC (36∶2) showed significantly higher intensities than their protonated forms in the livers of control as shown in Figure 3A. However, this pattern was not shown in the liver of HBx-null and wt HBV 1.2 mice. The phospholipid species PC (34∶2) and PC (36∶2) were also found in intact potassium cationized forms in the liver of HBV1.2 (HBx-), whereas PC (34∶1) was mainly found in intact protonated forms in the liver of HBV1.2 (wt). Although the adduct formation is related to ionization in MS which is not related to biological phenomena but it showed significant differences indifferent liver tissues (Mock, HBV1.2 (HBx-), and HBV1.2 (wt)).

We previously demonstrated that the decrease of potassium cationized adduct formation in the focal ischemic region is due to energy failure by a decrease in Na^+^/K^+^-ATPase activity in this region [25]. Additionally, decreased Na^+^/K^+^ pump activity was observed due to lower potassium ion concentration in a necrotic region [38]. The low potassium ion concentration may cause the relatively lower intensity of potassium cationized forms of PCs in the liver of HBV1.2 (wt). Although further comprehensive studies are necessary to delineate whether HBV infection regulates ATPase activity, our results suggest that the activity of (Na^+^/K^+^)-stimulated ATPase is associated with regulating physiological liver regeneration during HBV infection.

Recently, Singh *et al*. have reported that autophagy regulates the lipid metabolism and metabolic syndrome in the liver [39]. Interestingly, HBx was reported to induce autophagy, which is required for HBV replication [40], [41]. Thus, lipid alterations observed in our study may be associated with autophagy induced by HBx. Further studies on this issue will result in a better understanding of HBV-mediated liver diseases.

## Conclusions

In conclusion, we analyzed the altered phospholipids using MALDI-IMS in regenerating livers of HBV mouse models. In this study, we identified several PCs and related enzymes that showed significant expression changes in the livers of HBV infection and HBV-mediated regeneration defects. These results suggest that HBV infection particularly through the HBx protein which induces alterations in PC expression and these are potentially involved in HBV-mediated regeneration defects and liver diseases. Further studies are needed to define the specific role of these altered PCs on HBV-mediated liver pathogenesis.

## Supporting Information

Figure S1 **PCA analysis of the averaged MS spectra of phospholipids from liver tissue sections obtained from 3 different conditions in negative ionization mode.** (A) Typical MS spectra of liver tissue sections in 3 different conditions obtained from imaging mass spectrometry. (B) PCA analysis of the IMS data acquired in positive-ionization mode.
(TIF)

Figure S2 **MALDI images of phospholipids from the biological replicate in regenerating liver tissues after partial hepatectomy.** Alterations of phospholipid distribution showed similar tendency between biological replicates.
(TIF)

Figure S3 **MS/MS data of 17 phospholipid ions listed in** **Table 1** **.**
(TIF)
