# Supplementary figures and images for: Phosphatidylcholine Alteration Identified Using MALDI Imaging MS in HBV-Infected Mouse Livers and Virus-Mediated Regeneration Defects
Source: PLoS One. 2014 Aug 7;9(8):e103955. doi: 10.1371/journal.pone.0103955 (PMC4125171; doi:10.1371/journal.pone.0103955)

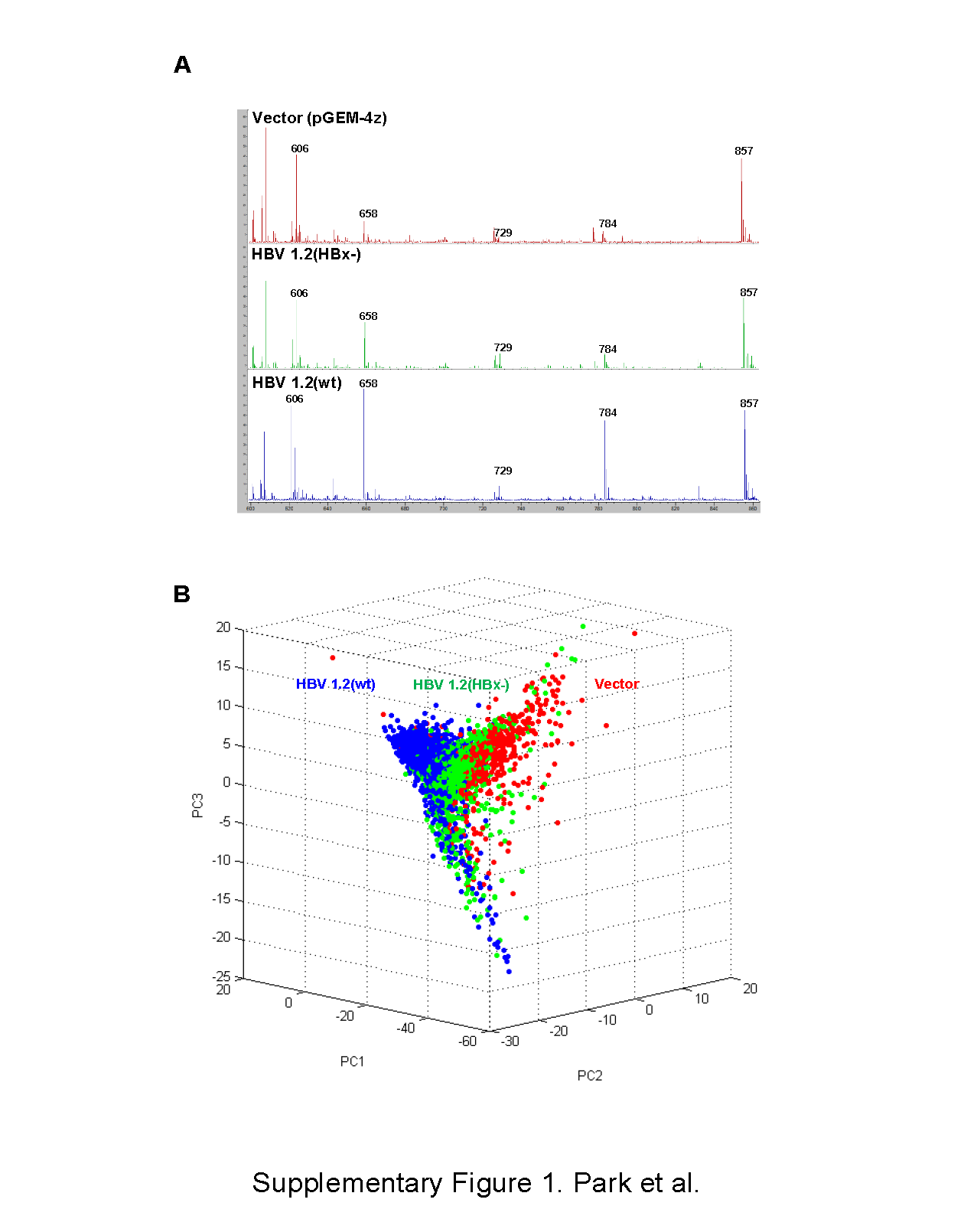

Supplement: Figure S1 — PCA analysis of the averaged MS spectra of phospholipids from liver tissue sections obtained from 3 different conditions in negative ionization mode. (A) Typical MS spectra of liver tissue sections in 3 different conditions obtained from imaging mass spectrometry. (B) PCA analysis of the IMS data acquired in positive-ionization mode. (TIF) [file pone.0103955.s001.tif]

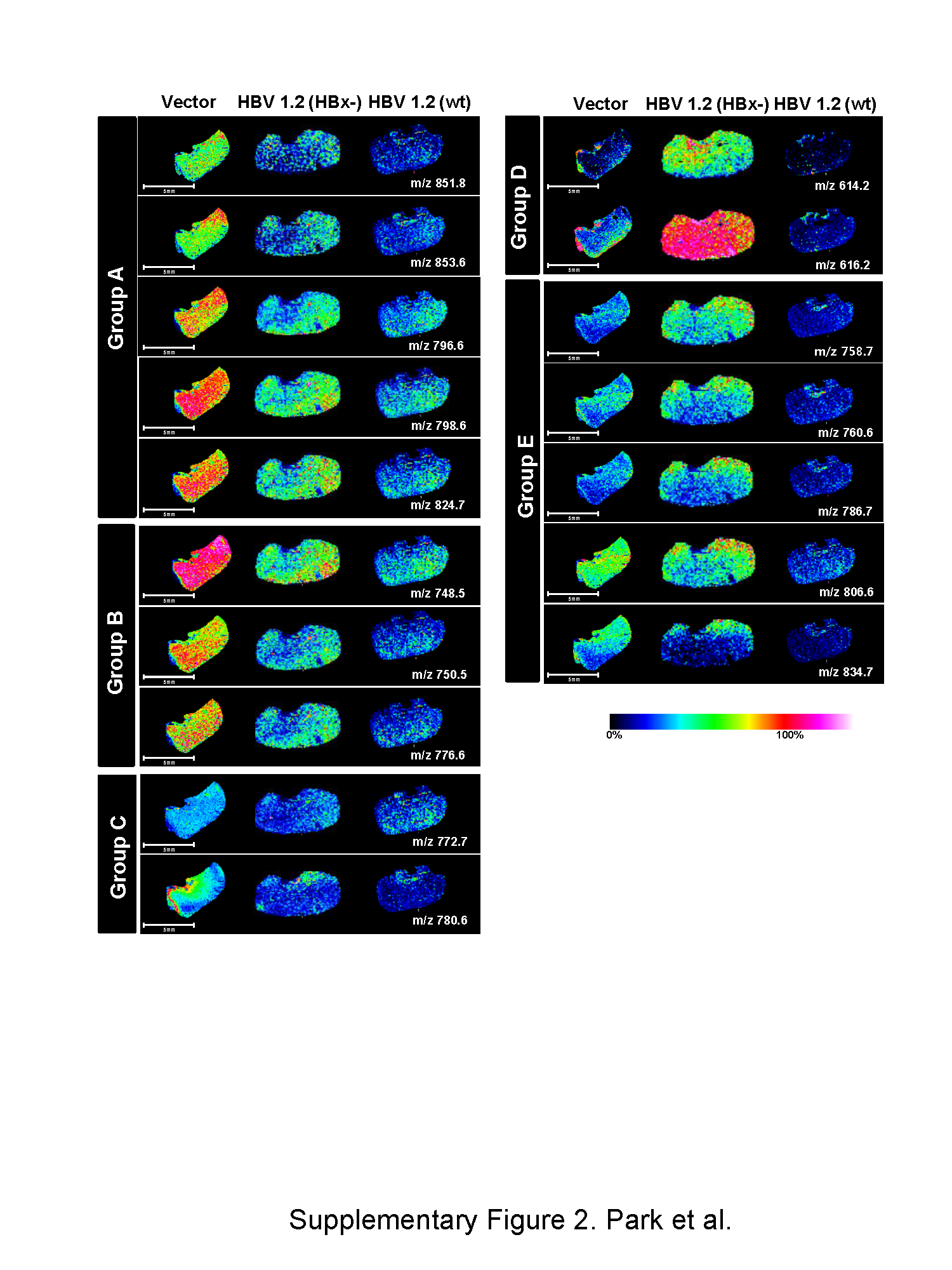

Supplement: Figure S2 — MALDI images of phospholipids from the biological replicate in regenerating liver tissues after partial hepatectomy. Alterations of phospholipid distribution showed similar tendency between biological replicates. (TIF) [file pone.0103955.s002.tif]

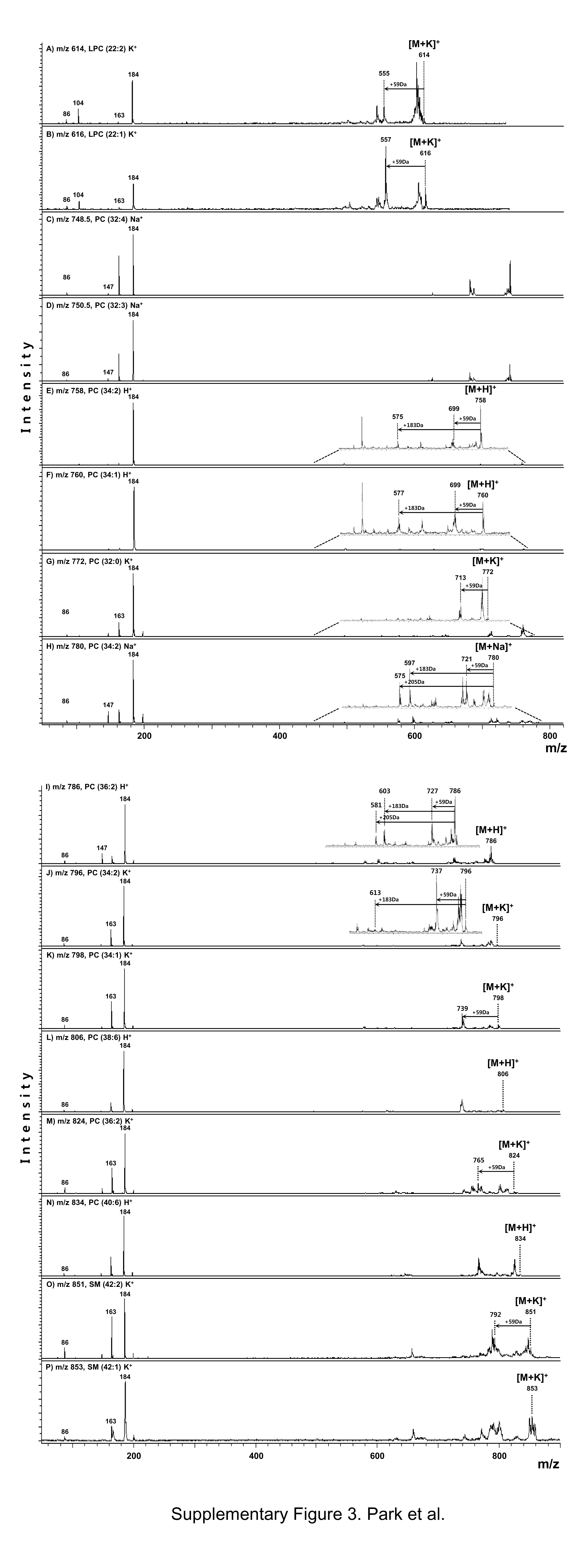

Supplement: Figure S3 — MS/MS data of 17 phospholipid ions listed in Table 1 . (TIF) [file pone.0103955.s003.tif]
